# Application of an Auditory-Based Feedback Distortion to Modify Gait Symmetry in Healthy Individuals

**DOI:** 10.3390/brainsci14080798

**Published:** 2024-08-09

**Authors:** Le Yu Liu, Samir Sangani, Kara K. Patterson, Joyce Fung, Anouk Lamontagne

**Affiliations:** 1 School of Physical and Occupational Therapy, McGill University, Montreal, QC H3G1Y5, Canada; 2 Feil and Oberfeld Research Centre, Jewish Rehabilitation Hospital Site of CISSS-Laval and Research Site of the Montreal Centre for Interdisciplinary Research in Rehabilitation (CRIR), Laval, QC H7V1R2, Canada; 3 Department of Physical Therapy and Rehabilitation Science Institute, University of Toronto, Toronto, ON M5G1V7, Canada; 4 KITE-Toronto Rehabilitation Institute, Toronto, ON M5G2A2, Canada

**Keywords:** neurologic gait disorder, audio feedback, stroke, young adults, walking

## Abstract

Background: Augmenting auditory feedback through an error-augmentation paradigm could facilitate the perception and correction of gait asymmetry in stroke survivors, but how such a paradigm should be tailored to individual asymmetry profiles remains unclear. Before implementing the paradigm in rehabilitation, we need to investigate the instantaneous effects of distorted footstep sound feedback on gait symmetry in healthy young adults. Methods: Participants (*n* = 12) walked on a self-paced treadmill while listening to their footstep sounds, which were distorted unilaterally according to five conditions presented randomly: small delay; small advance; large delay; large advance; or unmodified (control). The primary outcomes were swing time ratio (SWR) and step length ratio (SLR). Secondary outcomes included walking speed, bilateral swing time, step length, and maximum toe height, as well as hip, knee, and ankle angle excursions. Results: SWR (*p* < 0.001) but not SLR (*p* ≥ 0.05) was increased in all distorted feedback conditions compared to the control condition. Increased swing time on the perturbed side ipsilateral to feedback distortion was observed in the advanced conditions (*p* < 0.001), while swing time increased bilaterally in the delayed conditions (*p* < 0.001) but to a larger extent on the unperturbed side contralateral to feedback distortion. Increases in swing time were accompanied by larger maximum toe height as well as larger hip and knee joint excursions (*p* < 0.05 to *p* < 0.001). No differences in any outcomes were observed between small and large feedback distortion magnitudes. Conclusions: Distorted footstep sound feedback successfully elicits adaptation in temporal gait symmetry (SWR), with distinct modulation patterns for advanced vs. delayed footstep sounds. Spatial symmetry (SLR) remains unaltered, likely because auditory feedback primarily conveys temporal information. This research lays the groundwork to implement personalized augmented auditory feedback in neurorehabilitation.

## 1. Introduction

Motor learning is an important component of motor recovery following stroke [1]. Brain reorganization and cortical changes [2] are possible, especially with appropriate feedback [3] in the learning and recovery processes. Intrinsic feedback is provided by the internal sensory system that informs on one’s own movement, whereas extrinsic feedback can be provided by a therapist or a device [4]. Extrinsic feedback is particularly crucial when the intrinsic feedback system is impaired due to a brain lesion [5]. In neurorehabilitation, extrinsic feedback, such as verbal guidance during movement, can be effective in improving performance on simple motor tasks, but not in coordinated, multi-joint, and multi-limb movements such as those underlying gait symmetry [6,7]. Gait symmetry has not been shown to improve with conventional inpatient rehabilitation [8].

Rhythmic auditory cueing is an intervention extensively studied in gait training [9,10], e.g., an external auditory cue may be used to align foot strikes with auditory stimuli. Such a feedforward mechanism, involving synchronization between foot strikes and external cueing, is referred to as ‘entrainment’ [11]. Compared to the visual and tactile modalities, auditory cueing has the largest influence on gait parameters such as gait speed, stride time, stride time variability, etc. [12]. Auditory cueing can also be used to attenuate post-stroke asymmetry for short-term effects [13,14,15], but its application in motor learning requiring a longer period of training is still unknown. Besides auditory rhythmic cueing (feedforward), another approach is using auditory feedback as a source of real-time extrinsic feedback. Such an approach has not shown to alter significantly the stride length in different patient populations, such cerebral palsy, Parkinson’s disease, and stroke [16,17,18]. However, swing time and other temporal measures of gait symmetry were not examined in those studies. For people with stroke, Kim et al. (2021) used auditory feedback on weight bearing during walking and showed that the intervention, compared to conventional gait training, led to larger improvements in various spatiotemporal parameters such as paretic step time and non-paretic step length, as well as gait symmetry outcomes such as step length ratio, step time ratio, and single stance time ratio [19]. Auditory feedback is thus potentially beneficial to improve gait symmetry, but direct feedback on spatiotemporal aspects of gait has not been utilized and examined.

Feedback can be modified and distorted to enhance its effectiveness. The error-augmentation (EA) paradigm, which has mainly been studied in robotics, can be used to facilitate sensory-motor learning. Feedback is an essential basis for motor learning, and it is believed that artificially increasing the movement error would lead to faster learning [20]. The EA paradigm has been applied to visual feedback to reduce variation in weight shifting and postural sway among health young people, suggesting that EA could be used to improve balance and posture control [21,22]. The EA paradigm was also shown to be more effective than the error-reduction (ER) paradigm at improving gait asymmetry [23] in both healthy [24] and stroke [25] populations for gait outcomes such as step height, joints kinematics, and ankle trajectories.

A possible explanation of the mixed results observed regarding the effectiveness of auditory feedback on post-stroke gait symmetry is that people with stroke often have difficulty perceiving the presence and direction of their gait asymmetry [26]. The EA paradigm could potentially make the asymmetry more apparent and decipherable to the participants by increasing the extent of their asymmetry conveyed by the auditory feedback. It is important to recognize that post-stroke gait asymmetry varies in type (spatial, temporal, or both), direction (larger swing time on paretic side vs. non-paretic side), and magnitude [27,28]. Any application of an EA paradigm in this population would thus need to be personalized, and the potential change on spatiotemporal parameters needs to be studied among healthy individuals first. 

As a first step, we explored the use of distorted auditory feedback on footstep sounds in healthy young participants and examined the impact of such feedback on the alterations of gait symmetry. Our primary objective was to determine changes in step length and swing time ratios in response to distorted footstep sound feedback of different directions (delay vs. advance) and magnitudes (small vs. large) applied to either the right or left lower limb. Our secondary objective was to examine the associated changes in sagittal kinematics of the lower limbs. We hypothesized that (1) participants would increase or decrease their swing time unilaterally, or increase their swing time on one side while decreasing it contralaterally in response to the distorted feedback; (2) a large feedback distortion would lead to larger alterations in temporal-distances factors and gait symmetry in comparison to a small distortion; and (3) changes in swing time would be accompanied by changes in sagittal joint excursion at multiple joints (hip, knee, and ankle) and possibly changes in maximum toe height during swing.

## 2. Materials and Methods

### 2.1. Experimental Design

A cross-sectional, within-subjects design with repeated measures was used for this study.

### 2.2. Participants

Twelve (*n* = 12) healthy young adults, whose demographic characteristics are presented in Table 1, participated in this study. Participants were recruited based on the following criteria: (1) age between 18 to 35 years old, which corresponds to the young adulthood [29]; (2) having no self-reported auditory deficits; (3) having no conditions such as cardiovascular, respiratory, musculoskeletal, or other neurological conditions that could affect locomotion; (4) having no cognitive deficits indicated by a score < 25 on the Montreal Cognitive Assessment (MoCA). This study received ethics approval from the Research Ethics board of the Montreal Centre for Interdisciplinary Research in Rehabilitation (CRIR). All participants understood and signed the consent form before participating in the study.

### 2.3. Experimental Setup

The walking task was performed on a custom-built, self-paced treadmill (dimensions: 0.6 m × 1.5 m). The motor of the treadmill is driven by PID servo-control based on the real-time distance and velocity feedback obtained with a potentiometer tethered to the participant [30]; thus, the speed of the treadmill was instantaneously adjusted by the participant’s acceleration and deceleration.

**Figure 1:**
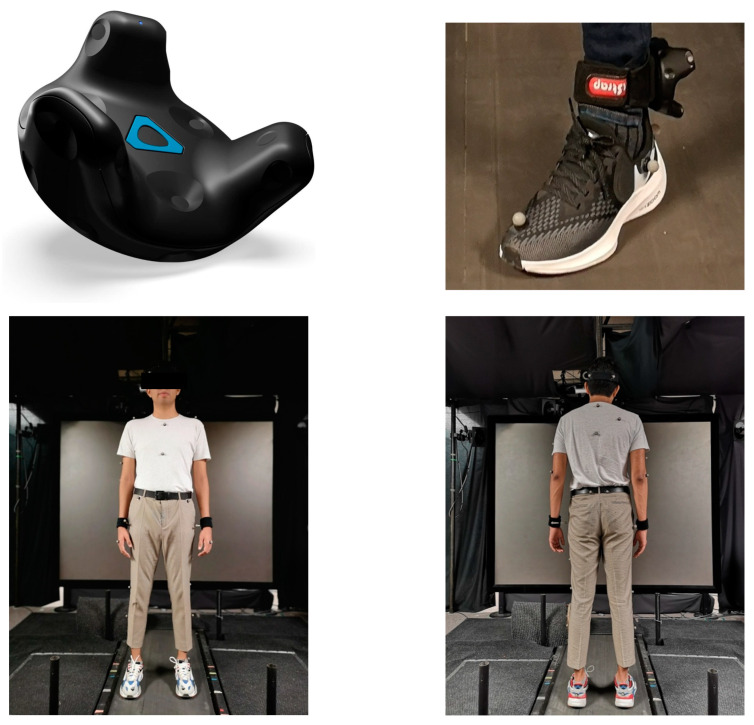
Top left: a tracker device from VIVE^TM^ used for the foot strike detection. Top right: the placement of the tracker device (around the ankle) during the experiment. Bottom: the 14 mm pearl hard markers placed on a participant based on the plug-in gait model (left: back view; right: frontal view). The participant is standing on a self-paced treadmill.

The footstep sound file used for the auditory feedback was created from an online sound effect library [31], and the same sound file was used for the footsteps on both sides. To control the timing of the footstep sound, a pair of VIVE^TM^ trackers (HTC Corporation, Taoyuan City, Taiwan) (Figure 1 top left) were strapped around each ankle of the participants’ (Figure 1 top right). A 2-camera VIVE motion capture system (HTC corporation, Taiwan) was used to capture the position of the trackers in real time with the Unity Engine (Unity Technologies, USA). Prior to the start of the walking trials, a baseline position of the trackers was established while the participant was standing in a static pose. During the walking trials, the footstep sounds would play based on a vertical threshold detection, i.e., when a foot would descend and the tracker would reach the pre-established threshold value, a trigger would be activated to play the footstep sound. The footstep sound was played via external speakers during the experiment and the volume was adjusted to a level at which participants would not be distracted by the environment noises (i.e., treadmill) or their natural footstep sounds. 

During the experiment, full body movements were recorded at 120 Hz in Vicon Tracker™ (Vicon, Oxford, UK) using a 6-camera system and 36 reflective markers (14 mm) placed on the participant’s specific body landmarks according to the Plug-in Gait model (Figure 1 bottom left and right).

### 2.4. Experimental Procedure

Prior to the start of the walking trials, the participants were habituated to the self-paced treadmill. The habituation lasted about 5 min or until the participants felt comfortable walking on the treadmill. The undistorted auditory feedback was also played during habituation, allowing participants to hear their undistorted (i.e., unmodified) footstep sound. This undistorted sound would serve as a reference for the participants when they were later exposed to distorted feedback. During the habituation period, the average swing time of each participant was calculated, and it was then used to compute delayed and advanced magnitudes described below.

There were five conditions of feedback that provided footstep sound on both sides, including four distorted feedback conditions and one control condition. In the distorted feedback conditions, footstep sound timing was distorted for the foot strikes on one side and undistorted on the other side. The four distorted feedback conditions were as follows: (1) a small magnitude of delay (sDelay) where a delay of 15% of the average swing time was added to the footstep sound; (2) a large magnitude of delay (lDelay) where the delay was 30% of the average swing time; (3) a small magnitude of advance (sAdvance) where the threshold value of sound detection was raised to a higher value (further from the floor) to simulate an advance of footstep sound by 15% of the average swing time; (4) a large magnitude of advance (lAdvance) where the threshold was raised further to simulate an advance of 30% of the average swing time. The control condition consisted of walking with undistorted footstep sound feedback of both sides. In a random order, the participants performed 18 walking trials, which consisted of two trials for the control condition and four walking trials for each of the four distorted feedback condition. Each distorted feedback condition had two trials where feedback was distorted on the left side and two trials on the right side. 

**Figure 2:**
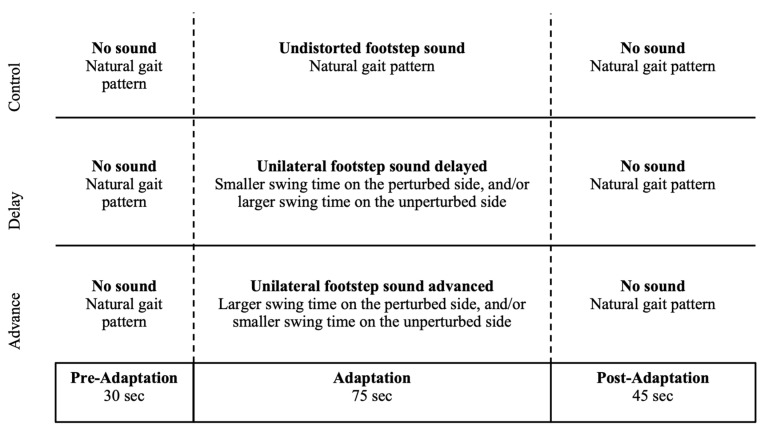
Feedback conditions and expected gait adaptations. Top row: the control condition where the auditory feedback is undistorted. Second row: the delay condition where the auditory feedback on one side is delayed. Third row: the advanced condition where the auditory feedback on one side is advanced. Last row: different phases of a walking trial and their duration.

Each walking trial consisted of three phases, which included 30 s of comfortable walking without the footstep sound presented (pre-adaptation phase), followed by 75 s of walking while listening to the footstep sound presented in one of the five conditions (adaptation phase), and finally 45 s of walking without the sound (post-adaptation phase). Before starting the trials, the participants were instructed to listen to the rhythm of their footstep sound when it appears and, if the sound is perceived to be distorted, to modify their gait pattern to make it sound symmetrical. However, if they perceived the sound to be undistorted as presented in the habituation, then they were not to make any gait change. When the footstep sound was absent, such as in the pre-adaptation and post-adaptation phases, they were to walk in their comfortable way. Participants were aware that the feedback could be distorted during the experiment, but they were not informed about the aspects related to the type and magnitude of the distortions. Sitting and resting for 5 min or more was allowed between the walking trials as needed. The experimental procedure, along with the expected gait pattern change according to the type of feedback distortion, is illustrated in Figure 2.

### 2.5. Data Analysis

The kinematic data were processed and exported from Vicon Nexus^TM^ 1.8.5 before being analyzed in Matlab™ R2020b. To avoid the effects of acceleration, data in the first 10 s of each trial were excluded while the rest of the trial was included for analysis. The data were dual-pass filtered with a 2nd order Butterworth filter at a cut-off frequency of 8 Hz, then normalized to 100% of the gait cycle.

The primary outcomes were swing time ratio (SWR) and step length ratio (SLR), which were used to measure the temporal and spatial aspects of gait symmetry, respectively [32]. The swing time was calculated as the time between the foot-off and foot-strike events of the same foot, which were computed using kinematic data from the markers placed on heels and first toes for each gait cycle, while step length was calculated by measuring the anteroposterior distance between bilateral toes at the foot strike. Ratios were calculated using the values of left and right leg with the larger value as the numerator [32], with a ratio of 1.0 indicating perfect symmetry. The secondary outcomes included were walking speed on the treadmill, bilateral swing time, step length, and maximum toe height, as well as sagittal hip, knee, and ankle angle excursions. For the purpose of analysis, the ‘left’ and ‘right’ sides were referred to as the ‘perturbed’ and ‘unperturbed’ sides, based on the side that the distortion was applied to. The means of all the outcome measures were obtained by averaging the values across gait cycles in each of the three phases (Pre-, Adaptation, and Post-).

### 2.6. Statistical Analysis

The data were found to deviate from a normal distribution, as revealed by the Shapiro–Wilk normality test; therefore, non-parametric tests were conducted. Generalized estimating equations (GEE), which do not require dependent variables to follow a normal distribution and were shown to be robust for smaller sample sizes [33], were used to compare SWR and SLR between the conditions (sDelay, lDelay, sAdvance, lAdvance) and across walking phases (pre-adaptation, adaptation, and post-adaptation). Outcomes of swing time, step length, and maximum toe height, as well as hip, knee, and ankle angle excursion, were also analyzed using a GEE model separately for the perturbed and the unperturbed side. When the GEE model revealed a significant effect, pairwise post-hoc comparisons were conducted with Bonferroni–Holm correction adjustments to account for the increased chance of type I error when conducting multiple statistical tests [34]. The statistical analyses were performed in SPSS v.24 (IBM Inc., New York, USA), and the alpha level was set at <0.05.

## 3. Results

**Figure 3:**
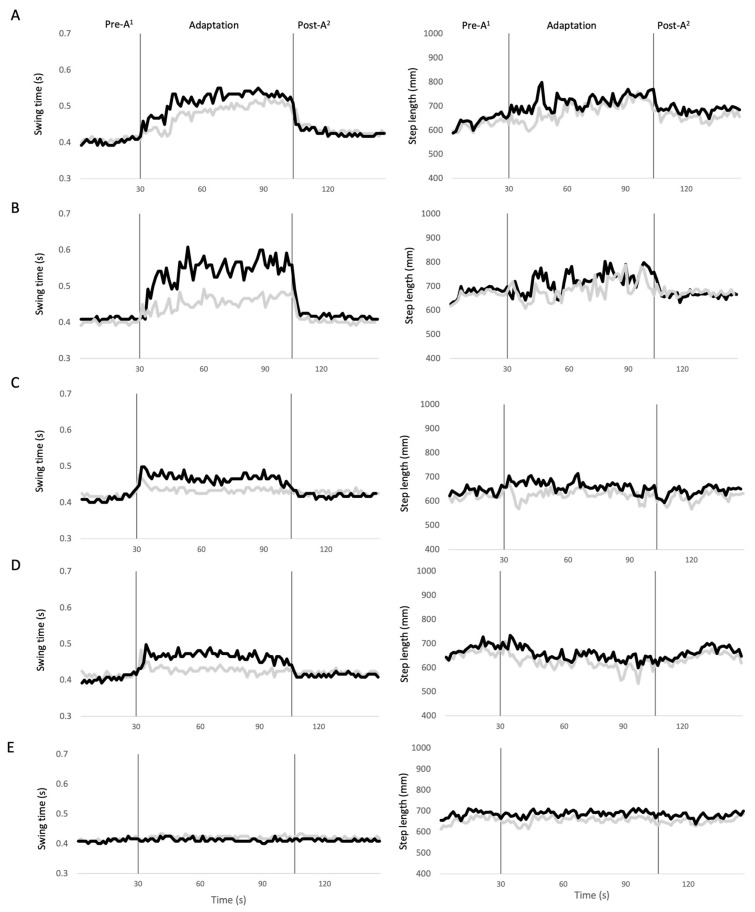
Traces of the bilateral swing time (left) and step length (right) of a participant over an entire walking trial in different conditions. Left and right sides are represented by the grey and black lines, respectively. Conditions are indicated by the letters, (**A**) small delay on the left side; (**B**) large delay on the left side; (**C**) small advance on the right side; (**D**) large advance on the right side; (**E**) control (undistorted footstep sound). ^1^ Pre-A: pre- adaptation, ^2^ Post-A: post-adaptation.

The average (±1 SD) delay for the sDelay and lDelay conditions were 0.08 ± 0.01 s and 0.17 ± 0.02 s, respectively. For the sAdvance and lAdvance conditions, the height thresholds for sound detection were raised by an average of 8 ± 2 mm and 14 ± 3 mm, respectively. All participants used the expected strategies in terms of swing time adaptation in all conditions illustrated in Figure 2. Figure 3 illustrates traces of bilateral swing time and step length of a representative participant with a distortion applied to only one side. Spatiotemporal adaptations were similar with respect to the perturbed and unperturbed sides regardless of whether the distortion was applied to the left or right lower limb. In both the sDelay (Figure 3A) and lDelay (Figure 3B) conditions, the participant showed a bilateral increase in swing time, but to a greater extent on the unperturbed vs. perturbed side during the adaptation phase. In the sAdvance (Figure 3C) and lAdvance (Figure 3D) conditions, only the swing time on the perturbed side showed an observable increase during the adaptation phase. As for the step length, none of the four distorted feedback conditions showed a noticeable increase or decrease on either side. In the control condition (Figure 3E), the participant maintained the same swing time and step length on both sides throughout the entire trial. In fact, the control condition did not show any significant change in SWR (Friedman test, χ^2^ = 4.67, *p* = 0.10) or SLR (χ^2^ = 4.67, *p* = 0.10) across phases.

### 3.1. Changes in Spatiotemporal Parameters

**Figure 4:**
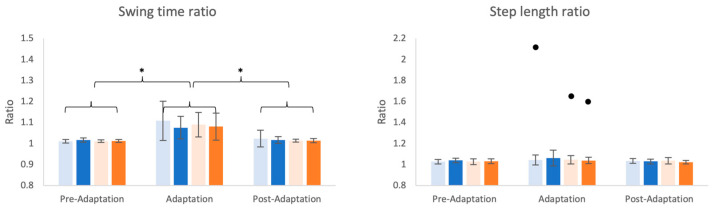
Spatiotemporal ratio of the four conditions across three phases, with swing time ratio on the left and step length ratio on the right. The conditions are indicated by different colors (light blue: small delay, blue: large delay, light orange: small advance, orange: large advance). Means and standard deviations are shown by the bars and whiskers. Circles above the bars represent the outliers. Statistically significant differences are indicated by * (*p* < 0.001).

A significant change in SWR (Figure 4, left) was observed between the phases (χ^2^ = 63.56, df = 2, *p* < 0.001) but not between the conditions (χ^2^ = 1.26, df = 3, *p* = 0.738). There were no interaction effects between phases and conditions (χ^2^ = 2.82, df = 6, *p* = 0.830). In the post-hoc pairwise analysis, the SWR in the adaptation phases, considering all four distorted conditions combined, was significantly increased compared to the pre-adaptation (mean of difference: 0.08 ± 0.01, *p* < 0.001) and post-adaptation phases (mean of difference: 0.07 ± 0.01, *p* < 0.001), while there were no significant differences between the pre- and post-adaptation phases (0.00 ± 0.01 *p* = 0.195). There was no significant change in SLR (Figure 4, right), however, due to feedback perturbation conditions (χ^2^ = 0.62, df = 3, *p* = 0.891) or phases (χ^2^ = 5.16, df = 2, *p* = 0.081) or the interaction between conditions and phases (χ^2^ = 5.09, df = 6, *p* = 0.532). Of note, this lack of significant effects existed despite one participant (shown as a single dot in Figure 4, right) showing high values of SLR compared to the rest of the group during the adaptation phase for the sDelay, sAdvance, and lAdvance conditions.

**Figure 5:**
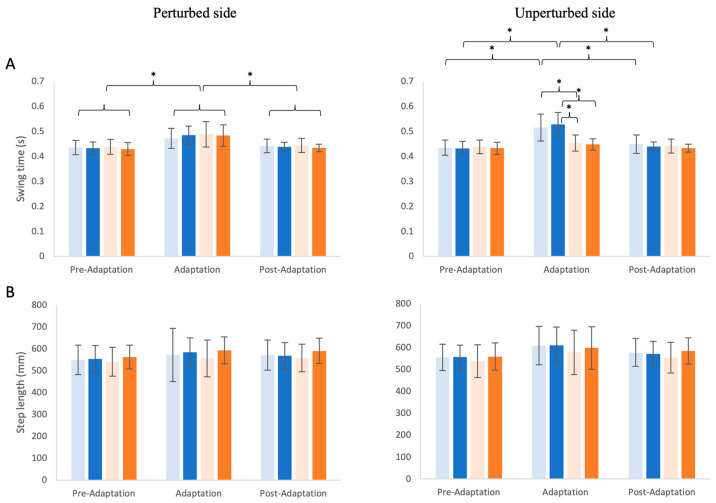
Bilateral swing time (**A**) and step length (**B**) of the four conditions across the three phases, with the perturbed side shown on the left and the unperturbed side shown on the right. The conditions are indicated by different colors (light blue: small delay, blue: large delay, light orange: small advance, orange: large advance). Means and standard deviations are shown by the bars and whiskers. Statistically significant differences are indicated by * (*p* < 0.001).

Similar to the SWR, the swing time of the perturbed side (Figure 5A left) was also modulated across phases (χ^2^ = 50.20, df = 2, *p* < 0.001), but it did not differ between perturbed feedback conditions (χ^2^ = 1.18, df = 3, *p* = 0.758) and showed no condition × phase interaction (χ^2^ = 1.28, df = 6, *p* = 0.973). Furthermore, the post-hoc analysis showed that the swing time of the perturbed side for all four distorted conditions combined was significantly increased in the adaptation phases compared to the pre-adaptation and post-adaptation phases (*p* < 0.001), but no difference was found between the pre-adaptation and post-adaptation phases (*p* = 0.263). The swing time of the unperturbed side (Figure 5A right), contrary to the perturbed side, was significantly altered by the conditions (χ^2^ = 29.48, df = 3, *p* < 0.001) and phases (χ^2^ = 61.27, df = 2, *p* < 0.001), with also a significant condition × phase interaction effect (χ^2^ = 34.28, df = 6, *p* < 0.001). In the post-hoc analysis, only the delay conditions (sDelay and lDelay) induced larger swing time on the unperturbed side in the adaptation vs. the pre-adaptation and post-adaptation phases (*p* < 0.001). Moreover, in the adaptation phase, the swing time of the unperturbed side for the delayed feedback conditions (sDelay and lDelay) were significantly larger (*p* < 0.001) than that of the advanced feedback conditions (sAdvance and lAdvance), and swing time was similar between the sDelay and lDelay conditions (*p* = 0.523).

The step length on both the perturbed side and unperturbed side remained unaffected by the feedback perturbation conditions (perturbed: χ^2^ = 4.34, df = 3, *p* = 0.227; unperturbed: χ^2^ = 5.81, df = 3, *p* = 0.121) and the phases (perturbed: χ^2^ = 3.88, df = 2, *p* = 0.144; unperturbed: χ^2^ = 4.41, df = 2, *p* = 0.110), with no significant condition × phase interactions (perturbed: χ^2^ = 0.48, df = 6, *p* = 0.998; unperturbed: χ^2^ = 3.31, df = 6, *p* = 0.769). The average speed (not shown on the graphs) across conditions ranged from 1.24 ± 0.21 m/s to 1.31 ± 0.18 m/s for the pre-adaptation phase, from 1.18 ± 0.25 m/s to 1.26 m/s ± 0.21 m/s for the adaptation phase, and from 1.26 ± 0.18 m/s to 1.36 ± 0.15 m/s for the post-adaptation phase. The walking speed was not changed significantly due to conditions (χ^2^ = 3.35, df = 3, *p* = 0.340) or phases (χ^2^ = 5.83, df = 2, *p* = 0.054), with no significant condition × phase interaction (χ^2^ = 0.28, df = 6, *p* = 0.999) effects.

### 3.2. Changes in Kinematic Outcomes

#### 3.2.1. Maximum Toe Height

**Figure 6:**
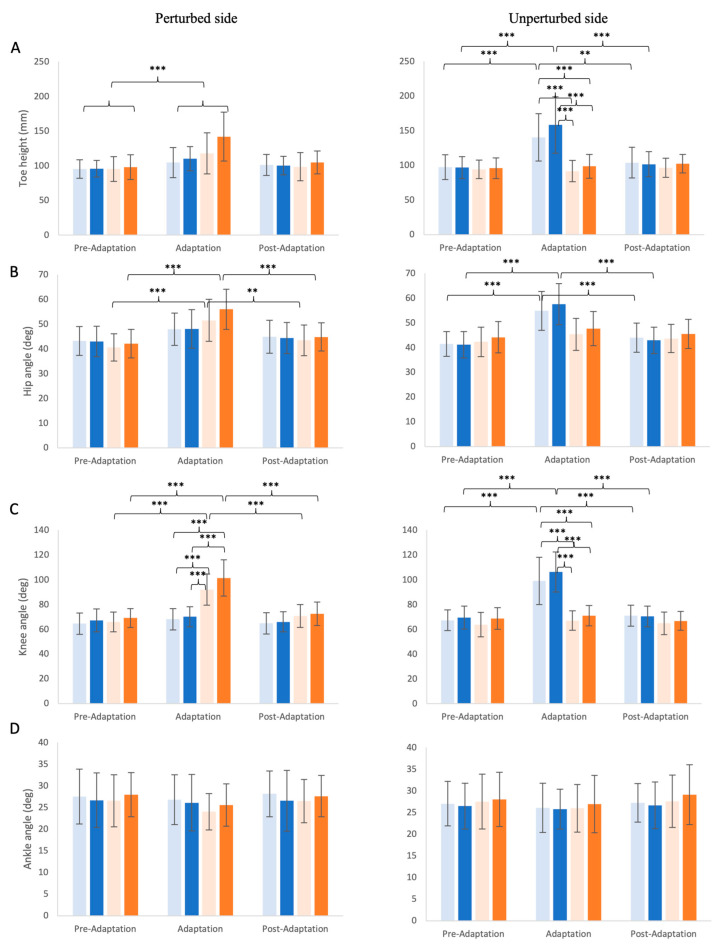
Bilateral maximum toe height (**A**), hip angle excursion (**B**), knee angle excursion (**C**) and ankle angle excursion (**D**) of the four conditions across the three phases, with the perturbed side shown on the left and the unperturbed side shown on the right. The conditions are indicated by different colors (light blue: small delay, blue: large delay, light orange: small advance, orange: large advance). Means and standard deviations are shown by the bars and whiskers. Statistically significant differences are indicated by ** (*p* < 0.01) *** (*p* < 0.001).

The maximum toe height on the perturbed side (Figure 6A, left) was significantly altered by the feedback perturbation conditions (χ^2^ = 10.16, df = 3, *p* = 0.017) and the phases (χ^2^ = 27.71, df = 2, *p* < 0.001), with no significant condition × phase interactions (χ^2^ = 4.34, df = 3, *p* = 0.227). Despite the significant main effect on the conditions, the post-hoc pairwise analyses did not show any significant difference between any condition pair (*p*-values ranged from 0.673 to 0.999). The maximum toe height in the adaptation phase was significantly increased compared to pre-adaption (*p* < 0.05), but not post-adaptation (*p* = 0.061). No difference was found between the pre-adaptation and post-adaptation phases (*p* = 0.656).

The maximum toe height on the unperturbed side (Figure 6A, right) followed a similar pattern as the swing time. i.e., significantly influenced by the conditions (χ^2^ = 36.58, df = 3, *p* < 0.001) and phases (χ^2^ = 33.22, df = 2, *p* < 0.001), with a significant interaction effect of condition × phase (χ^2^ = 36.99, df = 6, *p* < 0.001). Similar to the swing time on the unperturbed side, only the delayed feedback conditions (sDelay and lDelay) showed a significant increase of maximum toe height in the adaptation phase compared to the pre-adaption and post-adaptation (*p* < 0.001) phases. Furthermore, toe height in the adaptation phase for the delayed conditions (sDelay and lDelay) were significantly larger than for the advanced conditions (sAdvance and lAdvance) (*p* < 0.001). Toe height did not differ between sDelay and lDelay (*p* = 0.226).

#### 3.2.2. Hip, Knee, and Ankle Angle Excursion

Overall, the changes in sagittal joint excursion on both the perturbed and unperturbed sides were only present at the hip and knee joints, while ankle joint excursion remained mostly unchanged. Changes in hip and knee joint excursions also followed a similar pattern, in that they both increased on the perturbed side in the advanced feedback conditions and on the unperturbed side in the delayed feedback conditions. More specifically, significant main effects due to phase (χ^2^ = 42.70, df = 2, *p* < 0.001) and feedback condition (χ^2^ = 3.87, df = 3, *p* = 0.275) affected the hip joint excursion on the perturbed side (Figure 6B, left), whereas no condition × phase interaction (χ^2^ = 9.36, df = 6, *p* = 0.16) was present. The post-hoc analysis indicated that only the sAdvance and lAdvance conditions induced an increase in hip excursion in the adaptation phase compared to pre-adaption and post-adaptation phases (*p* < 0.001), with a similar trend, but not significantly different, between the sDelay (*p* = 0.052) and lDelay (*p* = 0.065) conditions.

As for the hip angle excursion on the unperturbed side (Figure 6B, right), there was a main effect due to phase (χ^2^ = 52.20, df = 2, *p* < 0.001) but not condition (χ^2^ = 7.22, df = 3, *p* = 0.065), with a significant interaction effect of phase and condition (χ^2^ = 24.09, df = 6, *p* < 0.01). Post-hoc analyses revealed that contrary to findings for the perturbed side, it was the sDelay and lDelay conditions that induced a significant increase in hip excursion angle during the adaptation compared to pre- and post-adaptation phases (*p* < 0.001).

The knee angle excursion on the perturbed side (Figure 6C left) was also significantly modulated by the conditions (χ^2^ = 62.47, df = 3, *p* < 0.001) and phases (χ^2^ = 76.16, df = 2, *p* < 0.001), but also showed an interaction effect of condition × phase (χ^2^ = 47.47, df = 6, *p* < 0.001). The post-hoc analysis revealed that only sAdvance and lAdvance induced an increase in the adaptation compared to the pre- and post-adaptation phases (*p* < 0.001). In addition, knee angle excursions for both sAdvance and lAdvance were larger than sDelay and lDelay in the adaptation phase (*p* < 0.001), but no significant difference was found between sAdvance and lAdvance (*p* = 0.082) conditions. The knee angle excursion on the unperturbed side (Figure 6C right) was also influenced by the conditions (χ^2^ = 68.22, df = 3, *p* < 0.001) and phases (χ^2^ = 75.24, df = 2, *p* < 0.001), with a significant phase × condition interaction (χ^2^ = 56.49, df = 6, *p* < 0.001). It followed a similar pattern with the swing time and maximum toe height on the unperturbed side as only sDelay and lDelay induced a significant increase in knee angle excursion in the adaptation phase compared to the pre- and post- adaptation phases (*p* < 0.001). Moreover, sDelay and lDelay induced significantly larger knee angle excursion than sAdvance and lAdvance (*p* < 0.001) conditions in the adaptation phase, whereas no significant difference was found between sDelay and lDelay (*p* = 0.301).

The ankle angle excursions on both the perturbed side (Figure 6D left) and unperturbed side (Figure 6D right) were not significantly affected by the conditions (perturbed: χ^2^ = 4.78, df = 3, *p* = 0.189; unperturbed: χ^2^ = 3.46, df = 3, *p* = 0.326) or the phases (perturbed: χ^2^ = 4.44, df = 2, *p* = 0.109; unperturbed: χ^2^ = 2.62, df = 2, *p* = 0.270), with no significant interaction effects (perturbed: χ^2^ = 0.72, df = 6, *p* = 0.994; unperturbed: χ^2^ = 0.34, df = 6, *p* = 0.999).

## 4. Discussion

This study examined, for the first time, the effects of distorted auditory feedback in the form of delayed and advanced footstep sounds on the gait symmetry of healthy young participants. Overall, all distorted auditory feedback conditions successfully elicited changes in temporal gait symmetry, as reflected by changes in SWR. Changes in kinematic outcomes, such as maximum toe height, as well as sagittal hip and knee joint angle excursion, were also observed. Once the distorted feedback was removed, the spatiotemporal and kinematic parameters of gait returned to the baseline values. Different magnitudes of distorted feedback were also tested and, in contrast to our hypothesis (2), no significant differences were observed between the small and large magnitudes of distortion.

The results of this study may guide the design of a future EA training paradigm that involves auditory feedback to improve post-stroke gait symmetry. Although our focus was not on diagnosing neurological disorders, gait symmetry outcomes such as SWR and SLR may serve as important biomarkers to differentiate different gait disorders and inform on intervention, such as a personalized dual-task paradigm combining auditory feedback with proprioceptive training in post stroke rehabilitation [7,35].

### 4.1. Temporal vs. Spatial Changes

Overall, although the delayed and advanced feedback conditions led to different adaptation strategies, their effects on the resulting swing time symmetry were similar, inducing an increase in swing time ratio in all distorted conditions. In the advanced feedback conditions, participants modified their swing time ratio by increasing the swing time on the perturbed side only, which was a possible strategy presented in hypothesis (1). This strategy of prolonging the swing time on the perturbed side allowed participants to delay the foot strike on that side for the bilateral footstep sound rhythm to become symmetrical. For the delayed feedback conditions, we hypothesized that participants would either decrease their swing time on the perturbed side only or decrease it on the perturbed side while increasing it on the unperturbed side. However, results showed that participants increased their swing time bilaterally, but to a greater extent on the unperturbed side. There could be two potential explanations: first, participants increased their swing time on the unperturbed side instead of decreasing it on the perturbed side because the latter always remained subjected to a perturbation (i.e., foot strike sound always delayed); second, the intuitive strategy for the participants was to perform some degree of adaptation on the perturbed side, as performed for the advanced feedback conditions, even if the adaptation strategy was not optimal. The differences in adaptation strategies among the advanced and delayed feedback conditions could suggest that the delayed auditory feedback was more cognitively demanding and challenging for participants to develop the right strategy. 

We also found that the step length ratio and the bilateral step length remained unaffected by all feedback conditions. Studies that examined the use of auditory feedback on foot strike have also shown a lack of effect on stride length, which is another measure of the spatial aspect of gait, although they did not examine the temporal parameters of gait [16,17,18]. In fact, it has been suggested that visual feedback facilitates learning of spatial aspects of movement, whereas auditory feedback facilitates learning of temporal aspects [36], because vision is precise in the perception of spatial information while hearing is precise in the perception of temporal information [37,38]. Sejdic et al. (2012) showed that auditory rhythmic cues had the greatest effect on temporal dynamics of gait of healthy participants compared to visual and tactile cues, but spatial parameters of gait were not examined [12]. Therefore, providing exclusively auditory extrinsic feedback, as in this study, would lead to changes in the temporal domain while leaving the spatial aspect of gait unaltered. 

Most of the kinematic outcomes (the maximum toe height, hip, and knee joint angle excursions) in this study followed a roughly similar pattern as the swing time, which was an increase on the perturbed side in the advanced feedback conditions and an increase on the unperturbed side in the delayed conditions, which agrees with our hypothesis (3). In fact, an increased swing time is normally associated with an increased hip and knee flexion, which would also contribute to elevating swing phase toe position. However, the ankle joint excursion remained unaltered by the provision of feedback. We believe that in the present case, changing ankle range of motion would not assist in prolonging swing time. Larger ankle plantarflexion, for instance, is associated with a greater push-off force in late stance, resulting in shorter swing time/increased cadence, longer step length, and faster walking speed [39]. The fact that ankle angle excursion remained unchanged in the present study is consistent with the lack of adaptation in walking speed or step length observed. Therefore, the observed kinematic changes were likely an adaptation with respect to the temporal and not spatial aspect of gait.

### 4.2. Magnitude of Distortion Does Not Matter

In contrast to our initial hypothesis, the spatiotemporal changes observed in the small and large magnitudes of feedback distortion were similar. A tentative explanation for this finding is that the magnitude difference between small and large perturbations might not be perceived by the participants and thus did not induce a significant disparity in the magnitude of the swing phase, which was the gait parameter that was found to be modified by the different feedback conditions. Crosby et al. (2021) conducted a study on creating a temporal gait asymmetry on healthy participants by putting a cuff weight on their non-dominant leg. Their results suggested that detecting the extent of asymmetry in gait was not an easy task, as most participants incorrectly estimated the magnitude of temporal asymmetry [40]. Thus, perceiving changes in the extent of asymmetry, as in the present study, may have proved to be challenging for the participants. Our results, however, also show that kinematic outcomes such as maximum toe height as well as hip and knee angle excursions tended to be larger for the large vs. small magnitude perturbations (advanced condition on perturbed side and delayed condition on unperturbed side), although not significantly different and not to an extent that led to changes in swing time. Thus, it cannot be ruled out that participants did implicitly perceive, at least to a limited extent, the perturbation magnitude. It is possible that a larger difference between the small and large distortion conditions would have been easier for the participants to detect, and thus would have led to more significant differences in lower limb kinematics and swing time adaptation, as well as resulting SWR.

### 4.3. Implications for Future Studies

This study has implications for the use of auditory feedback as part of an EA paradigm to improve gait symmetry in neurological populations such as stroke. First, the distorted auditory feedback as applied in this study only had an effect on the temporal aspect of gait (i.e., swing time and resulting SWR). Since individuals with stroke present with either or both swing time and step length asymmetry [8], the distorted feedback used in this study may only be applicable to improving swing time symmetry. Second, since people with stroke may have more difficulty perceiving the presence of asymmetry than neurotypical individuals [26], and their capacity to respond to distortion may be limited due to the sensorimotor impairment, their response to distorted feedback should be investigated. It is possible that a larger magnitude of distortion of auditory feedback may be needed to induce swing time adaptations in this population. Finally, as people with stroke generally present a larger swing time on the paretic side [41], we suggest applying the advanced feedback on the non-paretic side and/or applying the delayed feedback on the paretic side, in order to enhance the perception of swing time asymmetry, as an EA paradigm, to induce the desired swing time adaptation. In both scenarios, an increase in swing time in people with stroke would occur on the non-paretic side as it would be the unperturbed side in the delayed feedback condition and perturbed side in the advanced condition. While it seems more desirable for changes to take place on the paretic side, individuals with stroke actually present with shortened non-paretic swing time and prolonged paretic swing time compared to their healthy counterparts [42]. Increasing non-paretic swing time duration would thus not only minimize differences with respect to healthy individuals but it would also increase the time spent in single stance on the paretic limb, which in itself is a desirable outcome. It should be noted that distorted auditory feedback could be inconsistent with the proprioceptive and visual feedback, thus causing sensory incongruency, which warrants further investigation. 

### 4.4. Limitations

This study focussed on instantaneous adaptations to distorted auditory feedback in healthy young adults as a first step towards understanding potential applications in gait rehabilitation in patient populations with gait asymmetry. Such duration of exposure was sufficient to elicit changes in swing time symmetry and to identify the nature of the adaptations taking place cross the different conditions, but it may also explain the lack of after-effect observed in the post-adaptation period once the feedback was removed. The impact of longer and repeated exposure of distorted auditory feedback on the extent of SWR changes should be explored in future studies. This study was also limited to a homogenous sample of healthy young adults, which limits the generalization of findings to other age groups and patient populations. Further research is thus needed before such a paradigm can be applied to population such as stroke, who present different patterns and extents of asymmetry as well as variable capabilities in terms of sensorimotor function and rhythm perception. Finally, although all participants used the expected strategies in terms of swing time adaptation, this study did not assess the degree of symmetry in auditory feedback achieved by the participants.

## 5. Conclusions

In conclusion, distorted auditory feedback in the form of footstep sounds modifies bilateral swing time in healthy young adults, leading to different patterns of gait adaptation depending on whether the auditory feedback is delayed or advanced. The swing time ratio was also increased in all distorted feedback conditions, suggesting that manipulation of footstep auditory feedback could be an effective intervention to elicit changes in swing time symmetry. Results also revealed similar extents of gait adaptation between small and larger magnitudes of distortion. Larger differences in perturbation magnitude may be needed to induce a scaling effect in gait adaptations. Overall, the distorted auditory feedback examined in this study shows a potential to improve gait symmetry in patient populations, although further work would be needed to determine how to tailor the perturbations according to the asymmetry profile of the individual.

## Figures and Tables

**Table 1:** Participant demographics.

Healthy Young Adult (*n* = 12)	Mean ± 1 SD
Age (years)	26.4 ± 4.68
Sex (male/female)	5/7 *
Dominant side ** (right/left)	11/1 *
Height (cm)	171.1 ± 10.329
Weight (kg)	66.8 ± 18.44
Comfortable walking speed (m/s)	1.52 ± 0.20

* Ratio; ** Dominant side determined using the Edinburgh Handedness Inventory.

## Data Availability

Due to ethical restrictions, the data presented in this study are only available upon reasonable request from the corresponding author and after approval from the institutional review board.
